# Bone marrow-derived mesenchymal stem cell and simvastatin treatment leads to improved functional recovery and modified c-Fos expression levels in the brain following ischemic stroke

**DOI:** 10.22038/IJBMS.2018.29382.7100

**Published:** 2018-10

**Authors:** Gila Pirzad Jahromi, Alireza P Shabanzadeh, Mina Mokhtari Hashtjini, Seyed Shahabeddin Sadr, Javad Rasouli Vani, Javad Raouf Sarshoori, Jason Charish

**Affiliations:** 1 Neuroscience Research Centre, Baqiyatallah University of Medical Sciences, Tehran, Iran; 2 Electrophysiology Research Centre, Neuroscience Institute, Tehran University of Medical Sciences, Tehran, Iran; 3 Department of Physiology, School of Medicine, Tehran University of Medical Sciences, Tehran, Iran,; 4 Departmentof Biochemistry, Faculty of Medicine, Baqiyatallah University of Medical Sciences, Tehran, Iran; 5 Department of Anatomy, Faculty of Medicine, Baqiyatallah University of Medical Sciences, Tehran, Iran; 6 Genetics and Development Division, Krembil Research Institute, Toronto, ON, Canada

**Keywords:** Behavioral assessment, Bone marrow stromal cell, Brain, c-Fos, Ischemic stroke, Simvastatin

## Abstract

**Objective(s):**

The beneficial outcomes of bone marrow-derived mesenchymal stem cell (BMSC) treatment on functional recovery following stroke has been well established. Furthermore, 5-hydroxy-3-methylglutaryl-coenzyme A (HMG-CoA) reductase inhibitors have also been shown to increase neuronal survival and promote the movement of BMSCs towards the sites of inflammation. However, the precise mechanisms mediating the improved neurological functional recovery in stoke models following a combination treatment of Simvastatin and BMSCs still remained poorly understood.

**Materials and Methods:**

Here, an embolic stroke model was used to experimentally induce a focal ischemic brain injury by inserting a preformed clot into the middle cerebral artery (MCA). Following stroke, animals were treated either with an intraperitoneal injection of Simvastatin, an intravenous injection of 3 ×106 BMSCs, or a combination of these two treatments.

**Results:**

Seven days after ischemia, the combination of Simvastatin and BMSCs led to a significant increase in BMSC relocation, endogenous neurogenesis, arteriogenesis and astrocyte activation while also reducing neuronal damage when compared to BMSC treatment alone (*P*<0.001 for all). In addition, based on western blot analysis, following stroke there was a significant decrease in c-Fos expression (*P*<0.001) in the combination treatment group.

**Conclusion:**

These results further demonstrate the synergistic benefits of a combination treatment and help to improve our understanding of the underlying mechanisms mediating this beneficial effect.

## Introduction

Following a reduction in blood flow to areas of ischemia, factors such as unchecked neuronal depolarization, acidosis, free radical generation, inflammation, and an increase in intracellular calcium levels can all contribute to the ensuing neuronal death (1). Cerebral ischemia can also act as strong stimuli for the induction of the c-Fos proto-oncogene in the brain. Various studies, performed both *in vivo* and *in vitro*, indicate that neuronal death after ischemia is preceded by prolonged c-Fos induction (2), and that c-Fos can promote the induction of pro-apoptotic genes causing cell death (3). 

The use of bone marrow stromal cells (BMSCs) has been proposed as a useful source of multipotent lineage stem cells and as a potential beneficial therapeutic strategy following stroke (4-7). Following experimental stroke, the benefits of BMSC therapy may involve their capacity for providing an accessible supply of neurotrophic factors (8-10). In addition to these paracrine-mediated anti-apoptotic and antigenic effects, BMSCs also secrete extracellular matrix components, which contribute to an increased rate of healing in wounded regions (11-13). These effects help to explain some of the improved functional recovery observed in stroke models following BMSC treatment; however, additional work is warranted to both extend our knowledge of the underlying mechanisms by which BMSCs mediate their effects and to develop means towards improving the potential therapeutic effectiveness of BMSCs.It has been shown that the effectiveness of BMSC treatment can be enhanced by the additional administration of a dose of Simvastatin, which is an inhibitor of 5-hydroxy-3-methylglutaryl-coenzyme A (HMG-CoA) (14). Indeed, following experimental stroke in rats, the use of this combinatorial therapeutic strategy led to a significantly decreased infarct volume, a reduction in neuronal excitotoxicity and a reduction in cytotoxic edema, which together contributed to an improved functional outcome in comparison with BMSC monotherapy. However, there remain gaps in our knowledge regarding the cellular mechanisms leading to this additive effect on improved neurological function. We therefore investigated the effect of Simvastatin together with BMSC treatment on c-Fos expression levels. c-Fos is a proto-oncogene with a leucine-zipper region allowing for dimerization with c-Jun in order to form the sequence-specific activating protein (AP-1) DNA-binding complex(15, 16). Reports indicate that neuronal c-Fos levels increase dramatically following experimental focal or global cerebral ischemia (17-19), hemorrhage (20), and focal brain injury (21). Interestingly, up-regulation of c-Fos expression can also be observed in the brain shortly after peripheral administration of endotoxin, suggesting a potential link between immunological inflammatory processes and the elevation in c-Fos observed following the stroke (22). Here, we demonstrate that the combination of Simvastatin and BMSC treatments following stroke leads to a significant reduction in brain c-Fos levels. The combination therapy also led to increase in neurogenesis, angiogenesis and gliosis, which taken together help to improve our understanding of the factors contributing to the improved additive functional outcome after the stroke. 

## Materials and Methods

***Rat focal ischemia model***

The joint council of the Electrophysiology Research Centre (Neuroscience Institute; Tehran University of Medical Sciences) approved this study on 10/11/2012. All animal work was performed according to standards and protocols approved by the Animal Ethics Community of Tehran University of Medical Sciences. Adult male Wistar rats weighing 270-300 g (Pasteur Institute; Tehran, Iran) were obtained and housed at 3–4 rats per cage. Rats were kept at 22±2 °C under a 12:12 hr light/dark cycle with free access to water and food. Experimental procedures were performed during daylight hr under aseptic conditions.

A localized cerebral ischemia was created by closing the middle cerebral artery occlusion (MCAO) (14, 23, 24). Throughout the procedure and during recovery, body temperature was maintained at 37 °C using heating pads and heat lamps. Prior to surgery, anesthesia was induced using 3.0% halothane and during the surgery was maintained with 1.5% halothane (25).

Following anesthesia, a longitudinal incision (1.5 cm) was made in the ventral cervical skin along the midline in order to expose the right internal carotid artery, common carotid artery, and external carotid artery. A ligation was made in the distal portion of the external carotid followed by perforation.

PE-50 tubing was used to withdraw blood, which was kept for 2 hr at room temperature in order to form a clot. The clot was then divided into 20 mm segments. The clot was advanced approximately 17 mm into the internal carotid artery using a modified PE-10 catheter until it was located approximately 1–2 mm from the beginning of the MCAO. Following clot and catheter removal along with closure of the wound, the animal was allowed to recover. 

***Experimental designs***

After the surgery, animals were divided into two groups: i) rats sacrificed at 48 hr after MCAO, and, ii) rats sacrificed 7 days after MCAO. The animals in each group further subdivided into four smaller groups (n =4 or 8 per each subgroup for rats sacrificed at 48 hrs or 7 days following MCAO, respectively).

The control animals consisted of rats given an intraperitoneal (IP) injection of 1 ml vehicle one hour following embolization, followed by1 ml of phosphate-buffered saline (PBS) injected into the tail vain at 24 hr after MCAO. The Simvastatin alone group received one dose of Simvastatin (40 mg/kg IP) 1 hr after the embolization. The BMSC alone group received approximately 3 ×10^6^ BMSCs in 1 ml PBS by administering into a tail vein 24 hr after the embolization (8, 14, 25, 26). Finally, the combination group received one dose of Simvastatin (40 mg/kg IP) 1 hr after embolization, followed by 1 ml of 3×10^6^ BMSCs in PBS via tail vein injection 24 hr after MCAO.

We have previously established that one dose of Simvastatin at 40 mg/kg given at 1 hour after embolization together with a dose of BMSCs (3×10^6^ cells in 1 ml PBS) is sufficient to promote a significant decrease in neurological deficits, infarct volume, and brain swelling (14). As previously described, a 40 mg/ml stock of Simvastatin (Arasto Pharmaceutical Chemical Inc.) was prepared. BMSCs were extracted from 6-week old adult male Wistar rats and cultured. Immunocyto-chemistry was used for the positive identification of mesenchymal cells as previously described (14). As described previously, 5-bromodeoxyuridine (BrdU) incorporation was performed on BMSCs in culture media followed by injection of 1 ml of 3×10^6 ^BMSCs in PBS into a tail vein (14, 23). 

***Neurological functional tests***

A modified Bederson’s scale was used to evaluate functional recovery by scoring motor deficits both before MCAO and following MCAO at various time points: 24 hr, 48 hr and 72 hr and 7 days (14, 23, 27, 28). Adhesive-removal tests (14, 23, 26, 29) were performed prior MCAO and at 24 hr and 7 days post- MCAO, and were then scored by someone blinded to the experimental groups. 

***Histological analyses and tissue processing***

The rats were sacrificed 7 days after reperfusion. Animal perfusion was performed with 250 ml of 0.09% NaCl and then with 4% paraformaldehyde (PFA) in PBS (30). Post-fixation of the brain was performed by 4% PFA in PBS for 24 hr at 4 °C. The brain was cut into seven equally spaced coronal blocks (2 mm), and processed using a citadel 2000 tissue processor (Shandon) followed by embedding in paraffin wax. After sectioning, 5 µm slices were placed on glass slides and subjected to hematoxylin staining. Slide scanning was performed using Scan Jet (Hewlett-Packard), and image analysis was performed using Motic image processing software (31). The volume of the infract was calculated as previously described (14).

***Immunohistochemistry***

A paraffin block of the lesion (bregma -1 to +1 mm) was cut into 6 µm thick sections (32). Every tenth coronal section was used for immunohistochemistry staining. Antibodies were used against the followings: Alpha smooth muscle actin (a-SMA), a pericyte and smooth muscle cell marker (33-35) (1:800; Dako); Ki-67, a marker of proliferation (36) (1:300; Santa Cruz); neuron-specific enolase (NSE), a ɣ-subunit of enolase marker that is present in neurons (37) (1:100; Santa Cruz); glial fibrillary acidic protein (GFAP), an astrocyte marker (36) (1:250; Santa Cruz), and BrdU (1:100; Sigma-Aldrich) (38). 

***Immunohistochemically quantification***

In the ischemic boundary zone (IBZ), the diameter of arteries positive for α-SMA from five slides was measured and used to quantify the density of α-SMA-positive arteries. Each slide was broken down into 8 fields and digitized under a Leica CM E Light Microscope using an Infinity Lite camera interfaced with a Micro Computer Imaging Device (MCID). Vascular density was calculated by dividing the total number of vessels by the total tissue-area, giving the number of α-SMA-vessels per mm^2^. In order to assess BrdU-positive BMSC migration, cells with BrdU localized in the nucleus were counted throughout all 10 coronal sections.

In order to quantify reactive astrocytes, the percentage of tissue area that stained positive for GFAP-positive area was measured. In addition, subventricular zone (SVZ) cells immuno-reactive for Ki-67 were counted to assess cell proliferation. Ipsilateral brain sections were subdivided into eight fields, beginning in the SVZ and going up to the IBZ. Counting of immune reactive cells was performed using a non-biased digital setup. The extent of the tissue damage was assessed using NSE-labeling. Quantification of the number of neuroblasts (Ki-67), reactive astrocytes (GFAP) and injured neurons (NSE) were performed using the optical fractionator method.

***Western blot***

For western blotting, animals were killed 48 hr following MCAO, and cortical tissue samples were stored in liquid nitrogen until use. A 0.5 cm^2 ^block from the lateral area of the infarcted cerebrum was put in 400 μl SDS lysis buffer. Samples were homogenized and heated to 85 °C for 8 min followed by centrifugation (12000 rpm) to separate protein from the pelleted debris (39).

Western blots were incubated in 1% milk in TBS-T plus primary antibody overnight at 4 °C (anti-c-Fos; 1:200; Ab-cam). Washing was performed 3X 15 min in TBS-T, followed by secondary antibody incubation (1:10000; Dako REAL Envision, Rabbit/Mouse, HRP conjugated). Band optical density was normalized against the corresponding β-actin band.

***Statistics***

Results are presented as means±SEM. Intergroup means were compared using one-way ANOVA and Tukey’s *post hoc* test. The results for BrdU immunopositive cell numbers were calculated using an unpaired t-test. *P*<0.05 was considered significant.

## Results

***Combination treatment induces cell migration after stroke***

**Figure 1:**
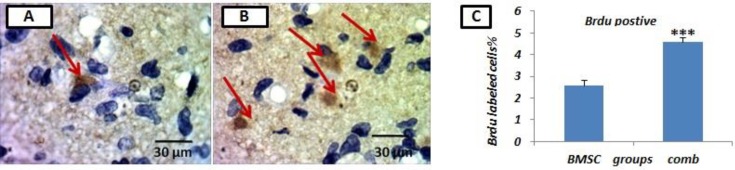
Staining of brain sections with 5-bromodeoxyuridine (BrdU) in groups of bone marrow stromal cells (BMSC) (A) or combination treated (B). Arrows display diaminobenzidine (DAB)-labeled cells (brown). (C) Graphs of the number of BrdU-positive cell numbers in the BMSC group in comparison with the combination treated (comb) group. Results based on means±SEM. ###* P*<0.001 versus BMSC group (n=4 per group)

The migration of BMSCs to the site of injury following stroke was determined by pre-incubating BMSCs with BrdU prior to injection. Following injection, the amount of BrdU positive cells in the peri-infarct region of the ischemic hemisphere was measured. Following MCAO, for rats treated with a combination of Simvastatin and BMSCs, the number of BMSCs labeled with BrdU was significantly enhanced in the lesion area when compared to rats treated with BMSCs alone (4.57±0.21/visual field versus 2.59±0.22/visual field, *P* <0.001). This indicates that the Simvastatin and BMSC combination therapy significantly increased the number of engrafted-BMSCs (Figure 1).

***Combination treatment leads to arteriogenesis, improved neurological function and reduced brain infarct volume***

**Figure 2:**
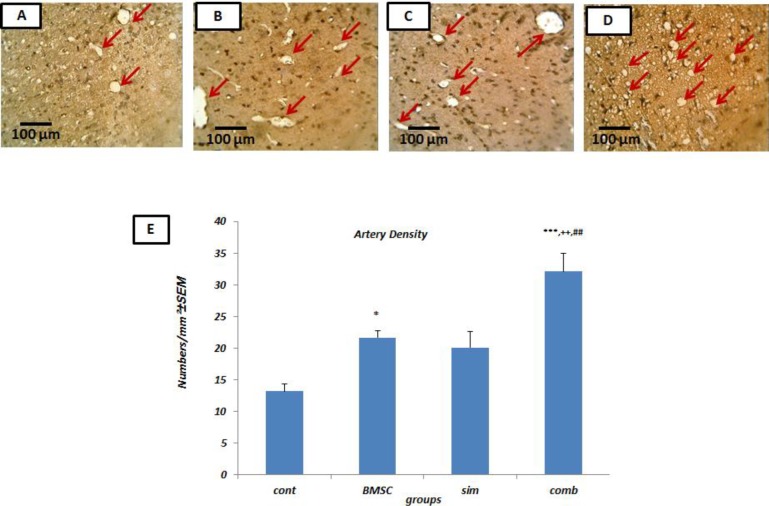
Increased arteriogenesis due to combining bone marrow stromal cells (BMSCs) and Simvastatin. Sample images of Alpha smooth muscle actin (α-SMA) immunostaining following middle cerebral artery occlusion (MCAO) from control (A), BMSC-treated (B), Simvastatin treated (C) and combination of Simvastatin and BMSC (D) groups. Arrows indicate diaminobenzidine (DAB)-labeled α-SMA-vessels (brown). (E) Arterial density quantification of control (cont), BMSC- treated (BMSC), Simvastatin- treated (sim), and combination-treated (comb) groups. Results based on means±SEM. * *P*<0.05, *** *P*<0.001 versus control group, ++ *P*<0.01 compare with Simvastatin group and ## *P*<0.01 compare with BMSC group (n = 4 per group)

The combination therapy significantly increased arteriole density (32.08±2.87 α-SMA-vessels/mm^2^) in comparison with controls (13.17±1.21a-SMA-vessels/mm^2^, *P*<0.001). The combination treatment was also more effective in increasing arteriole density when compared to the Simvastatin alone (20.07±2.52 α-SMA-vessels/mm^2^, *P*<0.01) and the BMSC alone treatment groups (21.69±0.99 α-SMA-vessels/mm^2^, *P*<0.01) (Figure 2).

**Figure 3:**
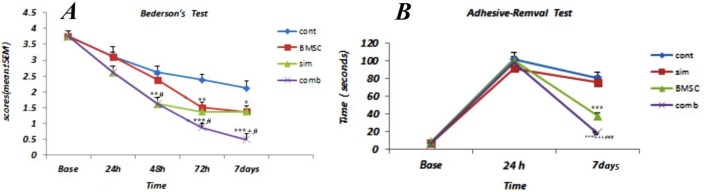
Neurological behavior after ischemic stroke. Neurological scores following stroke, based on the modified Bederson scoring system, for experimental groups (A). Adhesive removal test times at 24 hr and 7 days following stroke for experimental groups (B). Results are based on means ± SEM. * *P*<0.01, ** *P*<0.01, ***P<0.001 compare to control group, + *P*<0.05, ++*P*<0. 01, +++ *P*<0.001 compare to Simvastatin group, # *P*<0.5, ## *P* <0.01, ### *P*<0.001 compare to bone marrow stromal cells (BMSC) group (n = 8 per group)

Nerve function was measured prior to MCAO and post-MCAO at 24 hr, 48 hr, 72 hr, and 7 days in order to evaluate functional deficits following cerebral ischemia. At 24 hr after MCAO, similar motor deficits were observed in all animals. Beginning at 48 hr following MCAO, neurological scores as measured by Bederson’s scale were significantly improved in the combination treatment group (1.63±0.18) when compared to control rats (2.63±0.18, *P*<0.01) and BMSC alone treated rats (2.38±0.18, *P*<0.05), (Figure. 3). Evaluation of neurological scores at 7 days after ischemia also indicates significant improvement in functional outcome for the combination treatment as indicated by Bederson’s test (0.5±0.18) and adhesive-removal (23.87±1.83) tests, when compared to the controls (2.13±0.22, *P*<0.001 and 88.12 ± 4.91, *P*<0.001, respectively). Seven days after MCAO, the combination treatment group demonstrated an additive effect with significant differences observed in both Bederson’s test and adhesive removal tests when compared to the BMSC alone group (Bederson’s:1.38±0.18, adhesive-removal test: 43.75±4.55, *P*<0.05) and the Simvastatin alone group (Bederson’s:1.38±0.18, adhesive-removal test: 78.87±6.13, *P*<0.05) (Figure 3).

**Figure 4:**
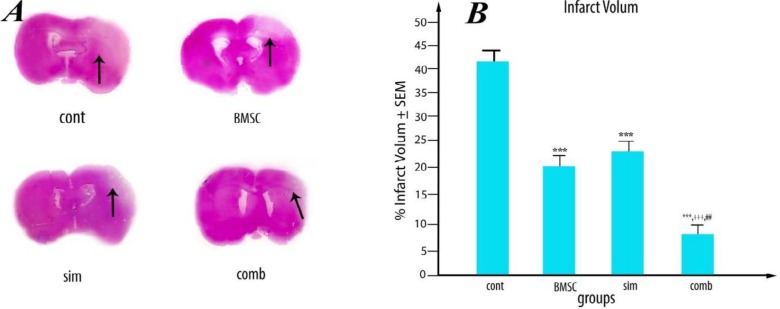
(A) Representative images of hematoxylin and eosin–stained coronal sections 7 days post- middle cerebral artery occlusion (MCAO) illustrating ischemic areas in experimental groups. (B) Quantification of infarct volume in the various treatment groups. Results are based on means±SEM. *** *P*<0.001 vs control, ++ *P*<0.05 compare to Simvastatin. # *P*<0.05 vs bone marrow stromal cells (BMSC) (n = 8 per group)

Hematoxylin and eosin histological staining results showed that the infarct volume of the combined-treatment group was considerably lower than those of control (41.81±2.37, *P*<0.001), Simvastatin (23.11± 3.13. *P*<0.01), and BMSC groups (19.88±1.56, *P*<0.05), indicating that the combination of Simvastatin and BMSC has an additive effect (Figure 4).

***Combination therapy stimulates astrocyte density following stroke***

**Figure 5:**
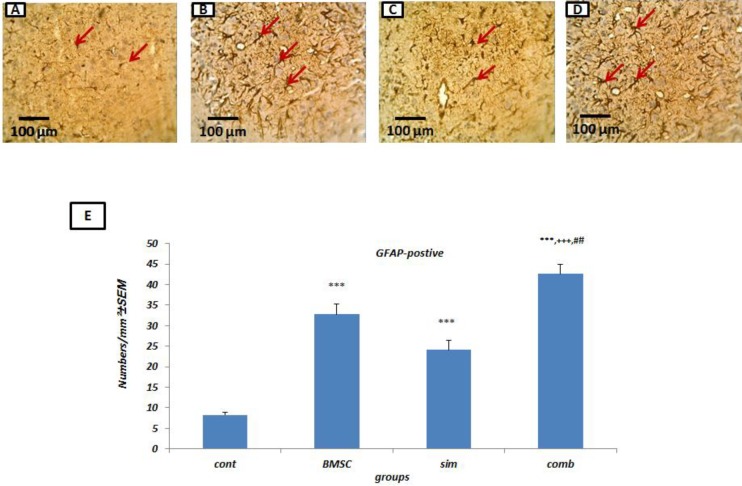
Increased astrocyte density following treatment with bone marrow stromal cells (BMSCs) and Simvastatin. Sample images of glial fibrillary acidic protein (GFAP) immunostaining in control middle cerebral artery occlusion (MCAO) rats (A), BMSC-treated (B), Simvastatin-treated (C) and combination-treated (D) rats.(E) quantifications of A-D (n = 4 per group). Results are based on mean±SEM. *** *P*<0.001 versus control rats, +++ *P*<0.001 compared to rats treated with Simvastatin and ## *P*<0.01 versus rats treated with BMSC. Arrows indicate diaminobenzidine (DAB)-labeled astrocytes (brown) located in the injury border zone. Asterisks indicate the infarcted zone at 7 days after MCAO

Astrocytes are essential for: i) adjustment of ions and neurotransmitters in the extracellular environment, ii) regulation of cerebral blood flow, iii) blood–brain barrier (BBB) permeability, and IV) the formation of glial scars after ischemic injury (40-42). Following the combination treatment, there was a significant increase in GFAP positive cells within the ischemic brain (42.60 ±2.35) when compared to the Simvastatin monotherapy (24.15±2.27,* P*<0.001), BMSC monotherapy (32.80±2.46,* P*<0.01) and controls (8.25±0.57,* P*<0.001) groups (Figure 5) (n= 4 per group).

***Combination treatment leads to an increase in the number of proliferating cells in the SVZ following stroke***

**Figure 6:**
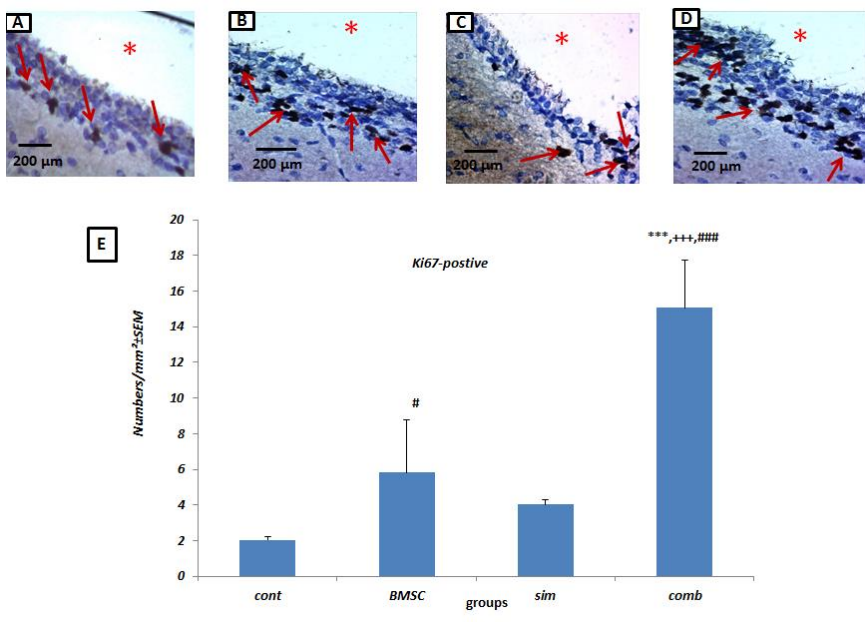
**I**ncreased numbers of proliferating cell in the subventricular zone (SVZ) of the ischemic brain following bone marrow stromal cells (BMSC) and Simvastatin combination treatment. Staining for Ki-67 in control (A), BMSC (B), Simvastatin (C) and combination (D) treated rats following middle cerebral artery occlusion (MCAO). (E) Quantification of data in A-D. Results are based on mean±SEM. * *P*<0.05 vs MCAO controls, *** *P*<0.001 vs MCAO controls, +++ *P*<0.001 vs Simvastatin group and ### *P*<0.01 vs BMSC group. Arrows indicate diaminobenzidine (DAB)-labeled proliferating cells (brown). The SVZ of the lateral ventricle in the injured hemisphere at 7 days after MCAO is shown with asterisks. (n= 4 per group)

The number of Ki-67 positive cells in the SVZ of the ischemic hemisphere was quantified to evaluate cell proliferation. As shown in Figure 6, the majority of Ki-67 immunoreactive cells were located along the SVZ, and the combination treatment resulted in an increase in the number of proliferating cells (14.11±1.52) when compared to control (2.03 ±0.19, *P*<0.001), Simvastatin alone (4.0.3 ±0.29, *P*<0.001) or BMSC alone (5.83 ±0.60, *P* <0.001) groups (Figure 6) (n=4/group).

***Combination treatment following stroke reduces neuronal injury and decreases c-fos levels in the ischemic brain***

**Figure 7:**
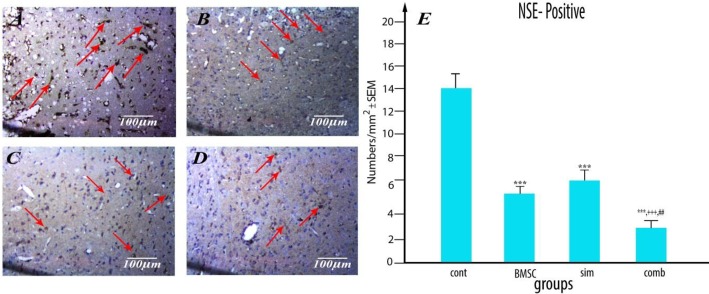
Decreased neuronal destruction following bone marrow stromal cells (BMSC) and Simvastatin combination treatment in middle cerebral artery occlusion (MCAO) rats. Neuron-specific enolase (NSE) staining in control (A), Simvastatin (B), BMSC (C) or combination-treated (D) MCAO rat groups. Arrows indicate diaminobenzidine (DAB)-labeled neurons (brown). (E) Plot of neuronal damage in the various treatment groups. Results are based on means ± SEM. *** *P*<0.001 vs control group, ++ *P*< 0.01 vs simvastatin group and # *P*<0.05 vs BMSC group (n=4 per group)

NSE, the ɣ-subunit of enolase, is present mainly in neurons and is regarded as a CNS-specific protein. This protein is normally cytoplasmic, and, therefore, elevated extracellular NSE concentration can be considered as an indicator of damaged cells. Here we demonstrated that treatment with Simvastatin and BMSC in combination markedly reduced mean NSE levels in the injured brain (3.400±0.25) compared to controls (17.14±1.03, *P*<0.001), Simvastatin-alone rats (8.25±0.74,* P* <0.001) and BMSC-treated rats (5.82±0.34,* P* <0.001), indicating that Simvastatin and BMSC show an additive effect (Figure 7) (n= 4 per group).

**Figure 8:**
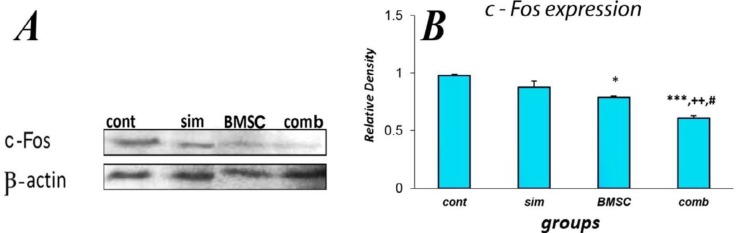
Decreased expression of c-Fos in the peri-infarct tissue in the bone marrow stromal cells (BMSC) and Simvastatin combination treatment groups following stroke. (A) Western blot analysis of brain samples for control (cont), Simvastatin (sim), BMSC or combination-treated rats (comb). (B) Quantification of expression level of c-Fos at 48 hr after embolization for the previously mentioned groups. For each sample, band intensity was normalized to β -actin. For each group data are presented as the mean of four separate brains±SEM. *** *P*<0.001 vs control, ++ *P*<0.01 vs Simvastatin and # *P*<0.05 vs BMSC (n=4 per group)

Finally, the effect of various treatments on c-Fos expression in the brain was assessed. c-Fos is a proto-oncogene expressed in the brain that can be involved in activation of immune pathological processes in the injured cerebral hemisphere and can be associated with behavioral impairment of functional ability (22, 43). c-Fos levels were assessed via western blots on brain lysates extracted from the infarcted right cerebral hemisphere. Rats receiving combination-treatment exhibited a noteworthy reduction in the relative mean c-Fos levels in the peri-infarct tissue (0.61±0.02) compared to the MCAO-alone controls (0.98±0.01, *P*<0.001), Simvastatin-monotherapy (0.88±0.5, *P*<0.01) or BMSC-monotherapy rats (0.79±0.01, *P*<0.05) at 48 hr following thromboembolic ischemic injury (Figure 8) (n = 4 per group).

## Discussion

Our results indicate that a combination therapy consisting of cellular and pharmacological agents can help to further facilitate neurobehavioral recovery in rats following stroke. There was significant improvement in reducing neurodegeneration following cerebral ischemia when combining Simvastatin with BMSCs compared to the individual monotherapies. Combination of Simvastatin and BMSCs furthermore promoted BMSC migration and enhanced endogenous arteriogenesis. This treatment approach therefore has potential to greatly reduce the pathophysiological consequences of cerebral ischemic injury under norm thermic conditions. Indeed, administration of the combination treatment reduced the volume of infarct and led to improvements in nervous function after MCAO compared to BMSC group. We demonstrated that the combination of Simvastatin with BMSCs can significantly decrease c-Fos levels following cerebral ischemia. Finally, we observed that the combination treatment enhances migration of BMSCs in the injured brain compared to BMSC alone treatment. Other recent studies have also noted BMSC migration to the sites of injury (44, 45) where they can potentially affect the environment through the secretion of trophic factors capable of stimulating neural repair and survival (46-48). However, it has been reported that there is a dose-dependent effect of these cells (49, 50), and that they have a relatively low capability for migration from the vascular system into tissues (50, 51). Thus, in order to augment their therapeutic effects, different strategies have been investigated to promote BMSCs migration towards the sites of injury. For example, stromal cell-derived factor-1 (SDF-1) and its receptor CXCR4 have been shown to affect BMSC migration towards the ischemic zone (52). Our study argues that Simvastatin, following a combination treatment, can be used to significantly increase the number of migrated-BMSCs in the site of the injury. One possible mechanism of this effect could be a Simvastatin-mediated increase in astrocyte-derived SDF-1α and vascular endothelial growth factor (VEGF) expression (53, 54).

Here, we also showed that the combination treatment can promote arteriogenesis as indicated by increases in the density of α-SMA-positive arteries in the ischemic brain when compared to control or Simvastatin/BMSC monotherapies. Simvastatin may therefore act as a protective factor through its effect on the promotion of angiogenesis and arteriogenesis, leading to improved microvascular reperfusion after stroke (55). Another study has also shown that Simvastatin can raise Ang1/Tie2 expression to induce vascular stabilization (56). 

Here, we also showed that combining Simvastatin with BMSCs after stroke promoted elevated reactive astrocyte levels in the ischemic brain and also increased cell proliferation in the SVZ when compared to MCAO control, Simvastatin and BMSC- monotherapy groups, which is in line with previous findings (28). Reactive astrocytes express various neurovascular trophic factors and chemokines such as SDF-1α, VEGF, glial cell derived neurotrophic factor (GDNF), and brain-derived neurotrophic factor (BDNF), which may positively contribute to neurogenesis, angiogenesis and neurite outgrowth following ischemia (54). This is line with our findings showing that the increase in reactive astrocytes as observed in the combination group was associated with reduced infarct size and improved functional outcome as measured by neurological scores (Bederson scale) and adhesive-removal tests. It has also been shown that Simvastatin reduces brain infarction and neurological deficits through reducing neuronal excitotoxicity and cytotoxic edema (57). Simvastatin can also potentially ameliorate oxidative stress responses, protect BBB integrity, and augment angiogenesis and neurogenesis following ischemia (54). Furthermore, Statins reduce stroke-induced peripheral immune depression (54).

To further elaborate on the means by which Simvastatin together with BMSCs help to additively promote neurological functional recovery, we investigated c-Fos levels, which have previously been shown to be significantly correlated with functional outcome (43) and with the expression of NSE in the peri-infarct region (37, 58). Studies have indicated that increased c-Fos expression may activate the immune pathologic processes of brain ischemia and may be associated with the behavioral changes and neurologic complications observed in neuronal injury (22, 43). c-Fos dimerizes with Jun family proteins to make the activator protein 1 (AP-1) transcription factor (15), and hence it can be considered as an important molecule involved in cell proliferation and differentiation (15). In addition to its roles in development and cell growth, c-Fos expression has also been implicated in apoptosis associated with non-proliferative conditions (59) or cellular injury (60). Indeed, in response to cerebral ischemia, it has been argued that neuronal c-Fos expression may promote the induction of apoptotic genes leading to cell death (16, 61, 62). This idea fits with the present findings, where the reduced c-Fos levels were observed in the combination treatment group following MCAO, and the same combination treatment was also associated with a number of improved molecular and functional outcomes. Interestingly, it has been previously shown that HMG-CoA reductase inhibitors can block the induction of c-Jun and c-Fos as mediated by platelet derived growth factor (PDGF) and Angiotensin II (63). Significant reduction of nuclear factor kappa B (NF-kB) and AP-1 activation in the kidney has also been observed following Statin treatment (64). Together, this is in agreement with our current and previous work demonstrating that BMSCs with Simvastatin leads to a reduction in infarct size 7 days post-MCAO (14). 

Finally, we also observed that Simvastatin increases BMSC migration into the target ischemic tissue, which may promote the elevation of beneficial secreted trophic factors (65), thus acting to augment intrinsic repair mechanisms in order to promote enhanced recovery of neurological functions.

## Conclusion

In this study, a combination treatment of Simvastatin and BMSCs led to a decrease in the neuropathological consequences of cerebral infarction following stroke. Given that, a significant reduction in c-Fos expression was also observed in this treatment group; this argues for the further identification and characterization of therapeutics that affect c-Fos expression. Ultimately, this will help towards the goal of ameliorating the devastating functional deficits arising due to cerebral stroke.
